# A promising PET tracer candidate targeting α‐synuclein inclusions

**DOI:** 10.1002/ctm2.1408

**Published:** 2023-09-07

**Authors:** Jie Xiang, Zhentao Zhang, Keqiang Ye

**Affiliations:** ^1^ Department of Neurobiology Fourth Military Medical University Xi'an China; ^2^ Department of Neurology Renmin Hospital of Wuhan University Wuhan China; ^3^ Faculty of Life and Health Sciences Shenzhen Institute of Advanced Technology Chinese Academy of Sciences Shenzhen China

1

Parkinson's disease (PD) is the most common neurodegenerative motor disorder. Its prevalence has increased dramatically. The clinical symptoms of PD overlap with other neurodegenerative disorders. Thus, its clinical diagnosis remains challenging. Although some biomarkers have been shown to improve diagnostic accuracy, it is hard to diagnose this disease at earlier stages without prominent and typical features. The characteristic pathology of PD includes loss of the dopaminergic neurons in the substantia nigra and accumulation of misfolded α‐synuclein in the remaining neurons. Visualization of dopaminergic neuron loss was a primary PET imaging strategy to diagnose PD. PET tracers have been developed to image dopamine synthesis, vesicular storage, release, reuptake, and dopamine receptors. However, these dopamine function‐related PET imaging are not very sensitive and unreliable to differentiation of parkinsonism disorders on a case‐by‐case basis.^1^, ^2^ In addition, patients who received medications may influence striatal uptake of these agents, limiting their reliability for measuring disease progression.^3^, ^4^ Thus, a small molecular PET radiotracer with high affinity and selectivity to fibrillar α‐synuclein could be helpful in quantifying the levels of α‐synuclein aggregation non‐invasively.

The α‐synuclein PET tracer would allow identifying prodromal and early‐stage synucleinopathies and can evaluate the progression of the disease. It can also help differentiate Parkinsonism caused by synucleinopathies, tauopathies, and other diseases. However, no radiotracers have been approved for clinical use in humans.^5^, ^6^ That is because the density of α‐synuclein aggregates in PD brains is lower than that of amyloid‐β (Aβ) and Tau in the brains of Alzheimer's disease (AD) patients. Thus, developing specific PET tracers for α‐synuclein is much more challenging.

To develop an α‐synuclein PET radiotracer, we searched the literature for its binding compounds and noticed that polyphenolic moiety in both dopamine (or polymers containing dopamine) and baicalein potently bind to α‐synuclein,^7^, ^8^, ^9^ suggesting that catechol groups may mediate binding activity toward α‐synuclein. Moreover, dopamine metabolites, such as melanin, possess a backbone similar to benzothiazine which is reported to bind with α‐synuclein fibrils.^10^ So we conjugate these two moieties via a connecting ethylene group to increase the chemical binding affinity and specificity toward α‐synuclein.

**FIGURE 1:**
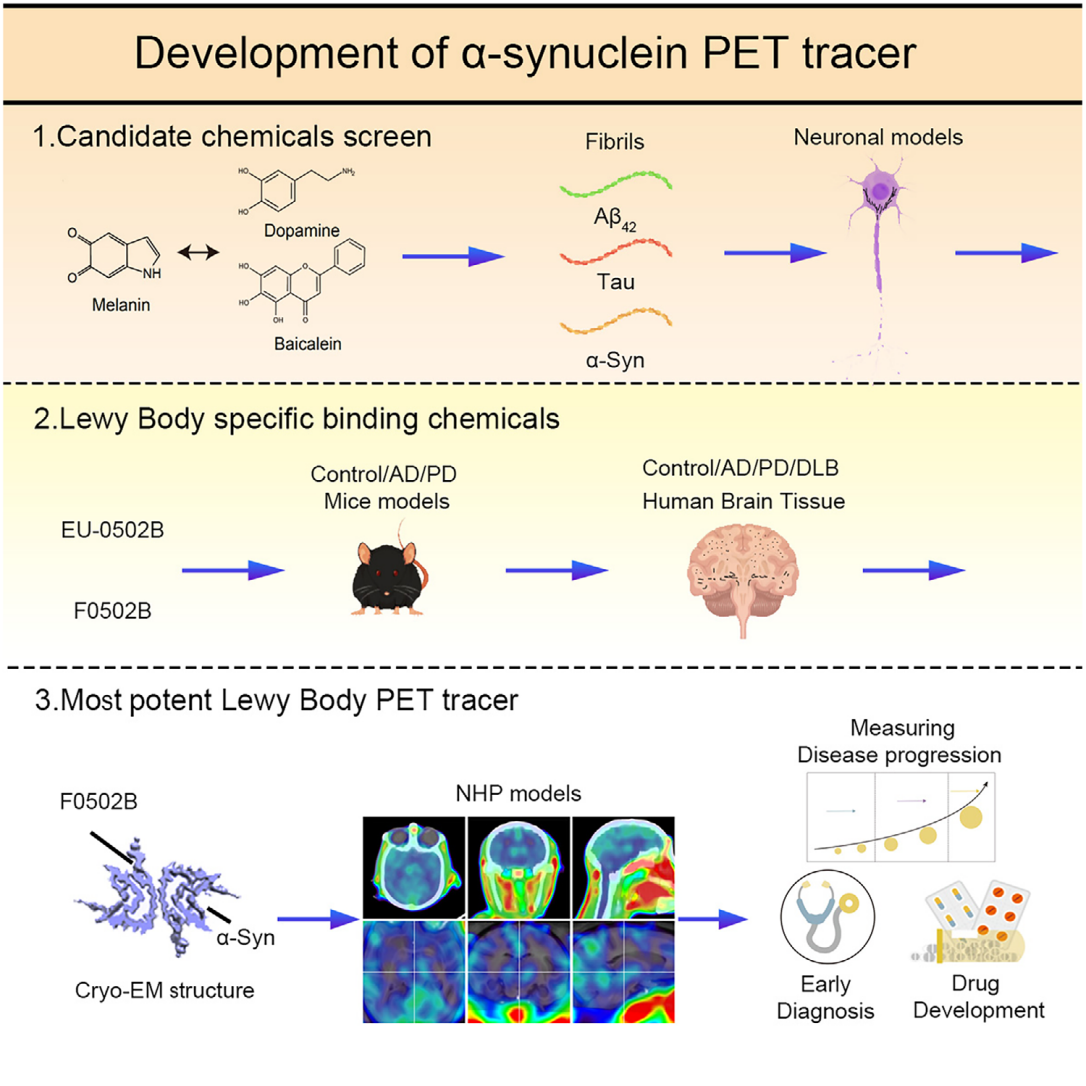
The screening strategy of α‐synuclein PET tracers. Reprinted with permission.

To find small compounds that specifically bind to α‐synuclein fibrils but not tau and Aβ fibrils, we performed the following experiments: (a) perform in vitro binding assays with pre‐formed fibrils (PFFs) to assess the selectivity of α‐synuclein; (b) apply animal models with misfolded aggregation pathology to test compounds specificity; (c) screen patient brain sections from synucleinopathies against AD; (e) determine the binding kinetics between α‐synuclein PFFs/ PD patient brain extracts and positive hits; (f) determine the brain permeability and wash‐out kinetics. (g) determine the in vivo PET images in non‐human primate models of synucleinopathies with the final candidate (Figure 1).

We screened commercial compounds, including catechol and benzothiazine groups, by performing an in vitro binding assay. Recombinant α‐synuclein, Aβ, and tau PFFs fibrils were co‐incubated with compounds and tested their binding affinities. Then we synthesized a series of derivatives by including methylamino or dimethylamino group on benzothiazole to increase the brain permeability and fluorescent signals. Then, we employed primary cultured neurons and mouse brain slides to verify their binding activities in vitro. We found that EU‐0502B reveals the strongest effect and selectivity with α‐synuclein. Notably, the carbonation of the phenol group eliminated its binding activity to α‐synuclein fibrils, underscoring its essential role in targeting α‐Synuclein fibrils. We labelled the N‐methyl group on EU05‐02B into the N‐2‐(2‐fluoroethoxy)ethyl‐N‐methyl group and named the newly synthesized derivative F0502B. Interestingly, substituting the hydroxyl group with the amino‐ or methylamino‐ group disrupted F0502B binding affinity to α‐synuclein fibrils but enormously elevated its interactions with Aβ or Tau fibrils. These structure‐activity relationship (SAR) studies emphasize that the phenol group is required for F0502B selective binding to α‐synuclein aggregates.

Our cryo‐EM structure of α‐synuclein fibrils in complex with F0502B further provides the atomic details for the binding between F0502B and α‐synuclein fibrils. We observed two stripes of additional and substantial densities accommodating in a deep groove of each protofilament in the complex fibril compared to the apo fibril, suggesting that these extra densities correspond to the F0502B ligand. F0502B inserts into a deep cavity on an α‐synuclein fibril surface with the phenol head residing inwards and the fluoro tail pointing outwards. F0502B repetitively aligns with α‐synuclein layers at a 1:1 ratio, establishing an intense bonding network along the fibril axis. In vitro, α‐synuclein fibrils and ex vivo multiple system atrophy (MSA) fibrils share a similar cavity for F0502B binding. This structure analysis explains that F0502B binds to α‐synuclein aggregation with high affinity and selectivity.

To characterize F0502B binding affinity and selectivity, ^18^F‐radio‐labelled F0502B was used in binding assays with recombinant and patients‐derived α‐synuclein, Aβ, and tau. The ^18^F‐F0502B binding α‐synuclein K_d_ value is 10.97 nM, much lower than its binding with Aβ (K_d_: 109.2 nM) and Tau (K_d_: 120.5 nM). The binding affinities are further elevated with PD or dementia with lewy bodies (DLB) patient‐derived a‐Syn fibrils with K_d_ 3.68 and 6.23 nM, respectively. To test ^18^F‐F0502B in situ binding selectivity, we applied autoradiography with brain slices from PD and AD patients. ^18^F‐F0502B can selectively bind with Lewy bodies in the striatum and substantia nigra region in PD but barely detect amyloid plaques or neurofibrillary tangles (NFTs) in AD. These findings suggest ^18^F‐F0502B can avidly bind to α‐synuclein fibrils in tissue sections.

The in vivo pharmacokinetics (PK) studies demonstrated that F0502B possesses favourable brain permeability and is swiftly washed out of the normal brain. To further verify the ^18^F‐F0502B diagnostic potential, we generated two PD non‐human primate (NHP) models by injecting α‐synuclein PFFs or adeno‐associated virus (AAV)‐α‐synuclein A53T into the striatum. In NHP, PET imaging shows that [^18^F]‐F0502B specifically recognizes α‐synuclein aggregates in the brains of monkeys. The promising results suggested it acts as a potential PET tracer for PD diagnosis.

Therefore, our study reveals a promising lead compound for imaging α‐synuclein and facilitating the diagnosis of synucleinopathies. These findings might provide a tool to improve the understanding of disease progression and potentially monitor the therapeutic efficacy in clinical trials.
